# Multidimensional Depressive Symptom Exposure and Incident Cognitive Decline: A Prospective Cohort Study

**DOI:** 10.1155/da/8846772

**Published:** 2026-08-06

**Authors:** Jun Guo, Shiyu Jiang, Chenxi Luo, Yarou Li, Sisi Guo

**Affiliations:** ^1^ Department of Nursing, Zunyi Medical University, Zhuhai Campus, No. 368, Jinwan District, Zhuhai City 519041, Guangdong Province, China, zmc.edu.cn

**Keywords:** cognitive decline, cumulative exposure, depressive symptoms, dynamic change, prospective cohort

## Abstract

**Background:**

Despite being a modifiable risk factor for cognitive decline, the influence of depressive symptoms on cognitive decline lacks a systematic assessment of its cumulative exposure intensity, temporal dimensions, and dynamic change.

**Aim:**

To systematically evaluate the multidimensional associations of depressive symptom exposure—including cumulative exposure, burden, duration, and dynamic change patterns—with incident cognitive decline, by adopting an analytical framework that integrates “static accumulation and dynamic evolution.”

**Methods:**

Among 8847 Health and Retirement Study (HRS) participants free of cognitive decline at baseline, cumulative depressive symptoms were quantified as the area under the curve (AUC) across 2012–2016 waves. A cut‐point was identified by maximizing the log‐rank statistic for cognitive decline risk within a clinically relevant range (2.5–3.5) and used to define cumulative burden and high‐exposure duration. Dynamic symptom patterns across three waves were identified, and their interaction with cumulative burden was examined. Multivariable Cox models estimated hazard ratios (HRs) and 95% confidence intervals (CIs) for incident cognitive decline risk associated with these exposure metrics.

**Results:**

During 3.67 median follow‐up years, 2311 incident cognitive decline cases occurred. Cognitive decline risk rose with escalating cumulative depressive exposure: the highest tertile (HR 1.42; 1.28–1.58), cumulative burden ≥0 (HR 1.34; 1.21–1.49), longest duration (HR 1.29; 1.12–1.48), and a progressively worsening trend (HR 1.52; 1.25–1.84) all significantly increased risk.

**Conclusions:**

Our findings demonstrate that the effect of depressive symptoms on cognitive decline is cumulative, time‐dependent, and dynamic. These results emphasize that sustaining a stable, low‐severity depressive symptom profile is essential for lifelong cognitive health.

## 1. Introduction

With the acceleration of global population aging, cognitive impairment has emerged as a defining public health challenge of the 21st century, placing a heavy burden on both individuals and society [1, 2]. Alzheimer’s disease and related dementias (ADRD)—including vascular, frontotemporal, and mixed etiologies—account for severe cognitive impairment in ~10% of US adults aged ≥ 65 years [1, 3, 4]. An additional 15%–22% live with mild cognitive impairment (MCI), a state of measurable cognitive decline with preserved activities of daily living that does not meet diagnostic criteria for dementia [1, 3, 4]. Cognitive function involves domains including complex attention, executive function, learning and memory, language, perceptual‐motor function, and social cognition. Decline in these domains (i.e., cognitive decline) is a core feature of ADRD and MCI [5].

The identification of modifiable risk factors and an in‐depth understanding of the mechanisms of their association are fundamental to the early prevention and delayed onset of diseases. Among numerous risk factors, a strong association between depression and cognitive decline has been well‐established by extensive epidemiological studies [6–10]. Individuals with depressive symptoms face a significantly elevated risk of cognitive decline, an association that remains independent of traditional confounders such as age, sex, educational attainment, and vascular risk factors [6–8, 11, 12].

However, a critical limitation pervades the existing literature, as the vast majority of evidence derives from cross‐sectional designs or single‐occasion depression assessments. Depression is intrinsically fluctuating and often chronic [13, 14], and its neuropathological impact on cognition—hippocampal atrophy, prefrontal hypofunction, and sustained neuroinflammation—accumulates progressively across the illness trajectory [15, 16]. A single snapshot can neither distinguish cumulative high‐intensity symptom burden nor capture the dynamic course of mood fluctuations over time; consequently, it may yield a biased—and potentially underestimated—assessment of cognitive decline risk. Integrating cumulative exposure intensity, high‐exposure duration, and dynamic change patterns of depressive symptoms may therefore offer a potentially useful framework for identifying the cognitive decline risk.

Drawing on prospective cohort data from the Health and Retirement Study (HRS), this study evaluates the associations of (1) cumulative depressive exposure, (2) cumulative exposure burden, (3) duration of high exposure, and (4) dynamic change patterns with the incidence of cognitive decline and further tests for potential interactions among these factors.

## 2. Methods

### 2.1. Data Source and Participants

We conducted this prospective cohort study in accordance with the Strengthening the Reporting of Observational Studies in Epidemiology (STROBE) statement [17]. Our data come from the nationally representative, biennial HRS of US adults, which since 1992 has collected comparable health, economic, and social data ideal for life‐course cognitive research [18, 19]. Ethical approval for the data used was granted by the University of Michigan Institutional Review Board, and all respondents gave signed consent after full disclosure.

**Figure 1:**
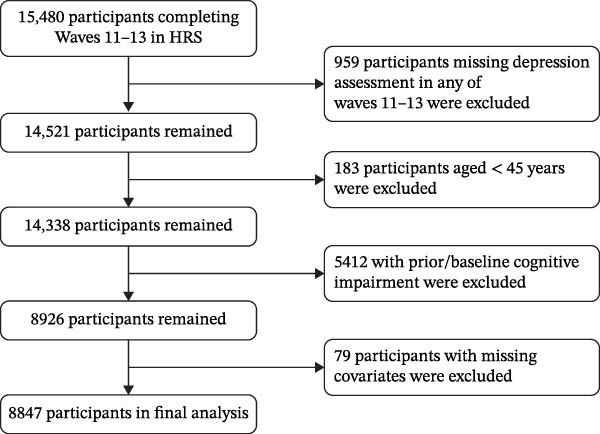
Flow chart of study participants.

We used waves 11–13 of the HRS (2012, 2014, and 2016) to construct a baseline cumulative depression score and then followed participants for two additional waves, with incident cognitive decline assessed in waves 14–15 (2018–2020). We excluded participants who (1) missed more than one of the three baseline depression assessments; (2) had cognitive decline or a history of memory‐related diseases at baseline; and (3) were younger than 45 years. In the HRS, 15,480 participants completed waves 11–13, and 8847 were finally included, as detailed in Figure 1.

### 2.2. Cumulative Depressive Symptom Assessment

In HRS, the 8‐item Center for Epidemiologic Studies Depression Scale (CES‐D) was employed (range 0–8), with a score ≥ 3 indicating depressive symptoms [20]. Higher scores denote more severe depressive symptoms. Cumulative depressive exposure was quantified by the area under the curve (AUC) across three CES‐D assessments collected every 2 years over a 4‐year span. AUC was computed as Σ (mean of two adjacent CES‐D scores × intersurvey interval), expressed in score years. This method has been widely applied to assess cumulative exposures [21–23].

### 2.3. Cognitive Decline Assessment

Cognitive function was assessed using cognitive tests that covered three domains—episodic memory (immediate and delayed word recall; 0–20 points), orientation (day, month, year, and week; 0–4 points), and executive function (a serial 7’s subtraction test; 0–5 points) [24, 25]. Domain‐specific raw scores were first standardized within eight age strata (45–49, 50–54, 55–59, 60–64, 65–69, 70–74, 75–79, and ≥80 years) derived from the baseline study sample. Cognitive decline was operationalized as performance >1.5 standard deviations below the age‐specific mean on any single domain [24, 25].

### 2.4. Covariates

Drawing on previous studies [20, 26], we considered the following candidate variables: demographic characteristics (age, sex, marital status, and education), lifestyle factors (smoking and alcohol consumption), and health status indicators (hypertension, diabetes, and sleep disorders).

### 2.5. Statistical Analysis

The study population was categorized into three groups based on the tertiles of the cumulative depressive exposure. Continuous variables were compared across groups using analysis of variance (ANOVA) or the Kruskal–Wallis test, as appropriate, while categorical variables were compared using the chi‐square test. The association between cumulative depressive exposure (low, medium, and high) and incident cognitive decline was evaluated using Cox proportional hazards regression models. Three models were constructed sequentially: a crude model (Model 1), a model adjusted for age and sex (Model 2), and a fully adjusted model that additionally included education, marital status, smoking, alcohol consumption, hypertension, diabetes, and sleep disorders (Model 3).

To identify the optimal cut‐point for cumulative average depressive symptoms, we employed an outcome‐oriented approach. Considering the established clinical threshold (CES‐D ≥3) [20], we prespecified a search range of 2.5–3.5 (centered at 3.0 and expanding 0.5 on each side). Candidate cut points were evaluated at 0.1 increments within this range. For each candidate, participants were categorized into high‐ and low‐exposure groups, and a log‐rank test was performed to compare cognitive decline risk between groups. The cut‐point that maximized the log‐rank chi‐square statistic (i.e., minimized the *p*‐value) was selected as the optimal cut‐point. To assess the stability of the identified cut‐point, we performed internal validation using bootstrap resampling with 500 iterations. In each iteration, we drew a bootstrap sample with replacement from the original cohort and repeated the cut‐point selection procedure (i.e., searching within the prespecified range of 2.5–3.5 at 0.1 increments and selecting the cut‐point that maximized the log‐rank statistic). On the basis of this cut‐point, a binary cumulative exposure burden variable (>cut‐point vs. ≤cut‐point) and an ordinal high‐exposure‐duration variable were constructed and entered into the aforementioned Cox proportional hazards models. High depression exposure duration was defined as the times of assessments with a high depression (over the cut‐point) among the three assessments, quantified as 0, 2, 4, and 6 years [22].

To assess the dynamic change patterns in depressive symptoms, participants were grouped into four categories according to the patterns of change observed over three assessments: “sustained improvement,” “initial improvement followed by worsening,” “initial worsening followed by improvement,” and “sustained worsening.” In Cox regression analyses, the “sustained improvement” category served as the reference to estimate the hazard ratios (HRs) of other change patterns for incident cognitive decline.

To test for a multiplicative interaction between cumulative exposure burden and dynamic change patterns, we added the product terms between the binary burden variable and the set of three dummy variables (representing the four‐category trend) to the Cox model. A likelihood ratio test (LRT) was performed to compare this full model against the nested model without the interaction terms.

As a sensitivity analysis, the Fine–Gray competing‐risks model—treating all‐cause mortality as a competing event—was employed to estimate subdistribution HRs for the association of (i) cumulative depressive exposure and (ii) high depression exposure duration with incident cognitive decline. To explore potential effect modifications, subgroup analyses were assessed across age, sex, and other key baseline characteristics. The LRT was used to evaluate the interaction terms between each subgroup variable and the three‐category cumulative exposure in the Cox models.

All analyses were performed using R software, Version 4.4.1. A two‐sided *p*‐value < 0.05 was considered statistically significant.

## 3. Results

### 3.1. Baseline Characteristics

Table 1 displays the baseline characteristics of the 8847 participants according to tertiles of cumulative depressive exposure. The mean age was 69.08 years, and 5246 (59.30%) were women. Compared with participants in the lowest tertile (T1), those with higher cumulative depressive symptom scores were more likely to be older, female, unmarried or without a partner, less educated, smokers, and abstainers from alcohol, and they exhibited a higher prevalence of hypertension, diabetes, and sleep disturbances (all *p*  < 0.001).

**Table 1:** Baseline characteristics of the participants according to tertiles of cumulative depressive symptoms.

Characteristic	Overall	T1 (≤ 1)	T2 (2–5)	T3 (> 5)	*p*‐Value
Number of participants	8847	3730	2574	2543	—
Age (years)^a^	69.08 (10.13)	65.76 (6.99)	71.94 (10.44)	69.83 (10.84)	<0.001
Sex^c^	—	—	—	—	<0.001
Male	3601 (40.70)	1021 (46.16)	1005 (45.43)	883 (39.92)	—
Female	5246 (59.30)	1191 (53.84)	1207 (54.57)	1329 (60.08)	—
Marital status^c^	—	—	—	—	<0.001
Married/partnered	5623 (63.56)	1719 (77.71)	1462 (66.09)	1350 (61.03)	—
Single/no partner	3224 (36.44)	493 (22.29)	750 (33.91)	862 (38.97)	—
Education^c^	—	—	—	—	<0.001
<High school	1156 (13.07)	141 (6.37)	262 (11.84)	323 (14.60)	—
High school/GED	2414 (27.29)	494 (22.33)	644 (29.11)	633 (28.62)	—
≥ Some college	5277 (59.65)	1577 (71.29)	1306 (59.04)	1256 (56.78)	—
Smoking^c^	—	—	—	—	<0.001
No	4133 (46.72)	1119 (50.59)	1065 (48.15)	1010 (45.66)	—
Yes	4714 (53.28)	1093 (49.41)	1147 (51.85)	1202 (54.34)	—
Drinking^c^	—	—	—	—	<0.001
No	3442 (38.91)	712 (32.19)	823 (37.21)	871 (39.38)	—
Yes	5405 (61.09)	1500 (67.81)	1389 (62.79)	1341 (60.62)	—
Hypertension^c^	—	—	—	—	<0.001
No	3566 (40.31)	1102 (49.82)	870 (39.33)	852 (38.52)	—
Yes	5281 (59.69)	1110 (50.18)	1342 (60.67)	1360 (61.48)	—
Diabetes^c^	—	—	—	—	<0.001
No	6701 (75.74)	1796 (81.19)	1685 (76.18)	1679 (75.90)	—
Yes	2146 (24.26)	416 (18.81)	527 (23.82)	533 (24.10)	—
Sleep disorder^c^	—	—	—	—	<0.001
No	7557 (85.42)	1972 (89.15)	1952 (88.25)	1899 (85.85)	—
Yes	1290 (14.58)	240 (10.85)	260 (11.75)	313 (14.15)	—
Cognitive decline^c^	—	—	—	—	<0.001
No	6536 (73.88)	1743 (78.80)	1680 (75.95)	1602 (72.42)	—
Yes	2311 (26.12)	469 (21.20)	532 (24.05)	610 (27.58)	—

Abbreviation: GED, general educational development.

^a^Mean ± SD.

^c^ *n* (%).

### 3.2. Cumulative Exposure of Depressive Symptoms and Incident Cognitive Decline

During a median follow‐up of 3.67 years (IQR 1.96–4.00 years) among 8847 participants, a total of 2311 incident cognitive decline cases (26.12%) occurred. The total follow‐up time was 27,232.7 person‐years, yielding an overall incidence rate of 84.86 per 1000 person‐years. The incidence rate of cognitive decline increased substantially with increasing cumulative depressive levels, ranging from 5.54 (95% confidence interval [CI] 5.17–5.94) per 1000 person‐years in the T1 group to 9.15 (95% CI 8.53–9.81) per 1000 person‐years in the T3 group. This trend remained significant after multivariable adjustment, with the HR for incident cognitive decline being 1.25 (95% CI 1.13–1.39) and 1.42 (95% CI 1.28–1.58) for T2 and T3 versus the T1 group, respectively (Table 2).

**Table 2:** Association of depressive symptoms with the risk of cognitive decline.

Exposure	Case, *n* (%)	Incidence rate ^∗^	Model 1	Model 2	Model 3
			HR (95% CI)	HR (95% CI)	HR (95% CI)
Cum. expos. (tertiles)					
T1 (*n* = 3730)	807 (21.6)	5.54 (5.17–5.94)	Reference	Reference	Reference
T2 (*n* = 2574)	701 (27.2)	7.50 (6.95–8.07)	1.32 (1.19–1.46)	1.32 (1.20–1.47)	1.25 (1.13–1.39)
T3 (*n* = 2543)	803 (31.6)	9.15 (8.53–9.81)	1.59 (1.44–1.76)	1.61 (1.46–1.78)	1.42 (1.28–1.58)
*p* for trend	—	—	<0.001	<0.001	<0.001
Cum. expos. (cont.)					
Per 1‐unit increase	2,311 (26.12)	7.07 (6.79–7.37)	1.03 (1.02–1.04)	1.03 (1.02–1.04)	1.02 (1.02–1.03)
Per 1‐SD increase	2,311 (26.12)	7.07 (6.79–7.37)	1.19 (1.14–1.23)	1.19 (1.15–1.24)	1.14 (1.09–1.18)
Cumulative burden					
< 0 (*n* = 7432)	1834 (24.7)	6.56 (6.26–6.87)	Reference	Reference	Reference
≥ 0 (*n* = 1415)	477 (33.7)	10.10 (9.22–11.00)	1.49 (1.35–1.65)	1.51 (1.36–1.67)	1.34 (1.21–1.49)
Exposure duration					
0 year (*n* = 6288)	1519 (24.2)	6.37 (6.05–6.70)	Reference	Reference	Reference
2 years (*n* = 1593)	460 (28.9)	8.16 (7.43–8.94)	1.25 (1.13–1.39)	1.27 (1.14–1.41)	1.18 (1.06–1.31)
4 years (*n* = 511)	183 (35.8)	10.90 (9.41–12.60)	1.65 (1.42–1.92)	1.66 (1.42–1.94)	1.46 (1.24–1.70)
6 years (*n* = 455)	149 (32.7)	9.82 (8.31–11.50)	1.49 (1.26–1.77)	1.51 (1.28–1.79)	1.29 (1.09–1.54)
* p* for trend	—	—	<0.001	<0.001	<0.001
Time course patterns					
Decrease–decrease	1152 (23.95)	6.29 (5.93–6.66)	Reference	Reference	Reference
Decrease–increase	504 (27.49)	7.64 (6.99–8.34)	1.19 (1.07–1.32)	1.19 (1.07–1.32)	1.13 (1.02–1.25)
Increase–decrease	539 (28.81)	8.05 (7.38–8.76)	1.26 (1.13–1.39)	1.25 (1.13–1.39)	1.18 (1.06–1.30)
Increase–increase	116 (35.15)	11.0 (9.06–13.2)	1.67 (1.38–2.03)	1.68 (1.39–2.04)	1.52 (1.25–1.84)

*Note:* Model 1: not adjusted. Model 2: adjusted for age and sex. Model 3: adjusted for age, sex, education, marital status, smoking status, drinking status, hypertension, diabetes, and sleep disorder.

Abbreviations: CI, confidence interval; HR, hazard ratio.

^∗^Incidence rate per 1000 person‐years.

The optimal cutoff point of cumulative average depressive symptoms associated with cognitive decline was 2.5. Bootstrap resampling (500 iterations) confirmed its stability (median: 2.5; 95% CI: 2.3–2.7). With this cutoff, the risk of incident cognitive decline increased when cumulative burden and duration of high exposure increased. Participants with cumulative burden ≥0 had a 34% higher risk of CVD (adjusted HR, 1.34; 95% CI 1.21–1.49) compared with those with cumulative burden <0 (Table 2). The incidence rate increased substantially from 6.56 per 1000 person‐years in the low‐burden group to 10.1 per 1000 person‐years in the high‐burden group. Participants with the longest exposure duration of high depressive symptoms (6 years) had a 29% higher risk of cognitive decline (adjusted HR, 1.29; 95% CI 1.09–1.54) compared with those with no exposure (Table 2). The incidence rate increased progressively from 6.37 per 1000 person‐years in the no exposure group to 9.82 per 1000 person‐years in the longest exposure group.

Dynamic change patterns of depressive symptoms were significantly associated with the cognitive decline risk. Compared with the sustained improvement pattern, the improvement‐worsening (adjusted HR, 1.13; 95% CI 1.02–1.26), worsening‐improvement (adjusted HR, 1.18; 95% CI 1.06–1.30), and sustained worsening (adjusted HR, 1.52; 95% CI 1.25–1.84) patterns were all associated with elevated cognitive decline risk (Table 2).

### 3.3. Joint Effects of Cumulative Depression Burden and Dynamic Change Patterns

The interaction between cumulative burden (high vs. low) and trend pattern (four categories) was assessed by a LRT (*χ* ^2^[3] = 8.28, *p* = 0.041), suggesting a potential synergistic effect (Table 3). The highest risk was observed in the high‐burden‐sustained worsening group (HR: 1.80; 95% CI: 1.33–2.43).

**Table 3:** Joint effects of depression patterns on cognitive decline risk.

Exposure	Case, *n* (%)	Incidence rate ^∗^	Model 1	Model 2	Model 3
			HR (95 CI)	HR (95 CI)	HR (95 CI)
Low_burden_decrease–decrease	1009 (22.8)	5.92 (5.56–6.29)	Reference	Reference	Reference
Low_burden_decrease–increase	406 (26.8)	7.35 (6.65–8.10)	1.22 (1.09–1.37)	1.21 (1.08–1.36)	1.17 (1.05–1.31)
Low_burden_increase–decrease	360 (27.4)	7.50 (6.75–8.32)	1.24 (1.10–1.40)	1.24 (1.10–1.40)	1.19 (1.05–1.34)
Low_burden_increase–increase	59 (33.5)	10.10 (7.71–13.10)	1.62 (1.24–2.10)	1.62 (1.25–2.11)	1.49 (1.15–1.94)
High_burden_decrease–decrease	143 (37.0)	11.20 (9.45–13.20)	1.80 (1.52–2.14)	1.83 (1.53–2.18)	1.59 (1.33–1.90)
High_burden_decrease–increase	98 (30.7)	9.14 (7.42–11.10)	1.49 (1.21–1.83)	1.50 (1.22–1.85)	1.33 (1.08–1.64)
High_burden_increase–decrease	179 (32.3)	9.42 (8.09–10.90)	1.54 (1.32–1.81)	1.54 (1.32–1.81)	1.36 (1.16–1.60)
High_burden_increase–increase	57 (37.0)	12.00 (9.09–15.60)	1.97 (1.51–2.57)	2.01 (1.54–2.62)	1.76 (1.34–2.30)

*Note:* Model 1: not adjusted. Model 2: adjusted for age and sex. Model 3: adjusted for age, sex, education, marital status, smoking status, drinking status, hypertension, diabetes, and sleep disorder. The interaction between burden and trend was tested by likelihood ratio test (LRT *χ* ^2^[3] = 8.28, *p* = 0.041).

Abbreviations: CI, confidence interval; HR, hazard ratio.

^∗^Incidence rate per 1000 person‐years.

### 3.4. Additional Analyses

In sensitivity analyses, we used the Fine–Gray competing‐risks model to evaluate the influence of all‐cause mortality on the associations of cumulative depressive symptoms, cumulative burden, duration of high exposure, and dynamic change patterns with incident cognitive decline, yielding largely unchanged results (Table S1). Using the established clinical threshold of 3.0 instead of the optimal cut‐point of 2.5 yielded highly consistent results, with HRs for cumulative burden and exposure duration remaining statistically significant and of similar magnitude (Table S6). Subgroup analyses indicated that the relationships of cumulative depressive symptoms, cumulative burden, duration of high exposure, and dynamic change patterns with cognitive decline were consistent across strata. Although only a few interactions reached statistical significance (Tables S2–S5), they did not alter the main conclusions.

### 3.5. Discussion

In this study, through a prospective follow‐up, we systematically assessed the associations between exposure intensity of depressive symptoms—including cumulative exposure and cumulative burden—and duration of high exposure and dynamic change patterns and the incidence of cognitive decline. The main findings include (1) a significant dose–response relationship between cumulative depressive exposure levels and the risk of cognitive decline; (2) cumulative burden of depressive symptoms, duration of high exposure, and dynamic change patterns were all independently associated with the risk of cognitive decline. These findings systematically reveal the multidimensional associations between depressive symptoms and cognitive decline through an integrated analytical framework of “static accumulation and dynamic evolution” addressing multiple dimensions such as exposure intensity, temporal dimensions, and dynamic changes.

Our study found a significant dose–response relationship between cumulative depressive exposure (calculated as the AUC) and the risk of cognitive decline. This pattern of progressively increasing risk remained consistent with multivariable‐adjusted models (Table 2), thereby supporting the “cumulative neurotoxicity” hypothesis [27]. Unlike single‐time measurements, our study utilized a longitudinal exposure indicator of depressive symptoms—namely, cumulative exposure—which more comprehensively captures the long‐term biological impact of depression as a chronic stressor on the brain. Persistently elevated depressive symptoms may lead to sustained dysregulation of the hypothalamic–pituitary–adrenal axis, resulting in glucocorticoid‐induced neurotoxicity [27, 28]. Concurrently, it may drive chronic neuroinflammation and exacerbate oxidative stress [29, 30]. The long‐term accumulation of these pathological processes could ultimately manifest as structural damage, such as hippocampal atrophy and accelerated cognitive decline [27–32]. The cumulative dose–response relationship revealed in this study provides epidemiological evidence for understanding the role of the long‐term cumulative effects of depressive symptoms in the onset and progression of cognitive decline.

This study also examined two key indicators of depressive exposure: cumulative burden (another indicator of exposure intensity) and duration of high exposure. The results demonstrated that both high cumulative burden and longer duration of high exposure were independent risk factors for cognitive decline, with adjusted HRs of 1.34 (95% CI: 1.21–1.49) and 1.29 (95% CI: 1.09–1.54), respectively. These findings reveal that the risk of depression‐related cognitive decline is determined not only by symptom severity but also by cumulative burden and exposure duration. Although the relationship between exposure duration and risk was nonlinear—a pattern that remained robust after accounting for the competing risk of death (Table S1)—the long‐term high‐risk exposure was unequivocally associated with the highest disease risk. This non‐monotonic pattern, where the 4‐year group showed a higher risk than the 6‐year group, warrants further consideration. This finding may reflect selective survival bias or differential dropout. Participants with longer (6‐year) depressive exposure who developed early cognitive decline or other adverse outcomes would have been excluded at baseline or censored. Additionally, those with chronic depression may have higher rates of loss to follow‐up due to depression‐related health issues or behavioral factors, and if these individuals had a higher underlying CI risk, their absence would underestimate the true risk in the 6‐year group. Furthermore, the smaller sample size and wider CI in the 6‐year group suggest less precise estimates. Alternatively, longer exposure might also mean longer treatment exposure; chronic depression may lead to more regular medical contact and antidepressant use, potentially mitigating cognitive risk. These possibilities warrant investigation in future studies.

Beyond static cumulative exposure, this study has further revealed that the longitudinal dynamic change patterns of depressive symptoms per se constitute an independent risk determinant for cognitive decline. Compared to the most favorable pattern of “sustained improvement,” any form of fluctuation—such as “initial improvement followed by worsening” or “initial worsening followed by improvement"—as well as a “consistently worsening” pattern, was associated with a significantly elevated risk of cognitive decline. This finding carries substantial clinical implications in that symptom instability may inherently reflect functional impairment or decompensation within brain emotion‐regulation circuits such as the prefrontal‐limbic system [33–35]. This neural vulnerability shares core neurobiological underpinnings with cognitive decline [35, 36]. Consequently, in clinical practice, monitoring the dynamic changes in depressive symptoms, rather than focusing solely on their severity at a single time point, is crucial for the early identification of individuals at high risk of cognitive decline.

The longitudinal analysis in this study confirms that the association between depressive symptoms and incident cognitive decline exhibits multidimensional and dynamically evolving characteristics. The cumulative depressive exposure (calculated via AUC), the cumulative burden, the duration of high exposure (defined by cut‐point), and dynamic change patterns collectively form a comprehensive risk assessment framework. These findings collectively establish a multidimensional framework integrating intensity, time, and change patterns, shifting the risk assessment of cognitive decline from a single‐point measure of symptom severity to a dynamic “cumulation–evolution” process. Based on this framework, this study has identified the highest‐risk subgroup of individuals exhibiting both a high cumulative burden and a persistently worsening symptom pattern, thereby providing new directions for early warning and intervention research.

Several limitations of this study should be acknowledged. First, the interaction between cumulative burden and trend was only marginally significant (*p* = 0.041) and did not survive correction for multiple comparisons (Bonferroni‐corrected *α* = 0.0071 for the seven comparisons in Table 3); therefore, the joint subgroup analyses should be interpreted as hypothesis‐generating rather than confirmatory, and replication in independent cohorts is warranted. Second, participants with more severe depressive symptoms or early cognitive decline may have been more likely to drop out during follow‐up, potentially introducing an informative censoring bias. Third, although our longitudinal study design and the exclusion of individuals with baseline cognitive decline partially mitigate concerns about reverse causation, the observed association may partly reflect prodromal cognitive decline—particularly in early mild cognitive decline—which can manifest as apathy, loss of interest, or subtle mood changes that resemble depressive symptoms, and distinguishing cause from consequence in observational data remains inherently challenging, so a bidirectional relationship cannot be fully excluded. Fourth, this study adopted a relatively lenient definition of cognitive decline (performance >1.5 standard deviations below the age‐specific mean on any single domain), a choice informed by the DSM‐5’s 1–2 SD range for neurocognitive disorders [5] and consistent with prioritizing sensitivity in large‐scale epidemiological studies [24, 25]; had a more stringent definition been used (e.g., decline in ≥2 domains or >2.0 SD), the estimated prevalence would be lower, and the association might be attenuated. Similarly, the optimal cut‐point of 2.5 for cumulative average depressive symptoms was derived from the HRS cohort using an outcome‐oriented approach centered on the clinical threshold of 3.0; although bootstrap resampling supported its internal stability, and sensitivity analyses using the clinical threshold of 3.0 yielded highly consistent results, generalizability to other populations requires external validation as such thresholds may be influenced by sample characteristics and the prespecified search range. Fifth, several unmeasured factors warrant discussion: we did not adjust for continuous baseline cognitive function (though excluding prevalent cognitive decline substantially reduces this concern); physical activity and social engagement may act as mediators rather than confounders, so adjusting for them could lead to overadjustment bias; and apolipoprotein E (APOE) genotype is largely independent of depressive symptoms, making substantial confounding unlikely. Finally, the assessment of both depressive symptoms and cognitive decline relied on rating scales that, though widely validated, remain susceptible to measurement error; our covariates (e.g., smoking, alcohol use, and sleep disorders) were self‐reported, subject to recall and social desirability bias; the median follow‐up of 3.67 years was sufficient to capture medium‐term risk, but long‐term outcomes require longer tracking; and as the study population consisted of US middle‐aged and older adults, caution is warranted when generalizing conclusions to other populations. Future research should employ objective measures (e.g., neuroimaging and biomarkers), extend follow‐up duration, and validate the model’s generalizability across diverse populations.

This prospective cohort study established that the intensity of depressive symptoms—encompassing cumulative exposure and burden—and their temporal dynamics, including duration and dynamic change patterns, are independently and multidimensionally associated with the incidence of cognitive decline. This study suggests that monitoring the long‐term progression of depressive symptoms at the individual level is crucial for the early warning and precise prevention of cognitive decline.

**Nomenclature**
CI: Confidence interval
HRS: The Health and Retirement Study
AUC: The area under the curve
CES‐D: Centre for Epidemiologic Studies Depression Scale.

## Author Contributions

Jun Guo contributed to the study conception and design. Jun Guo wrote the main manuscript text. Jun Guo, Shiyu Jiang, Chenxi Luo, Yarou Li, and Sisi Guo participated in the material preparation, data collection, and analysis. Jun Guo made significant contributions to revising and finalizing the manuscript.

## Acknowledgments

We are deeply grateful to the participants and interviewers of the Health and Retirement Study (HRS), the Gateway to Global Aging Data team for providing harmonized data, and the HRS for its open‐access, high‐quality datasets. HRS is conducted by the Institute for Social Research at the University of Michigan with funding from the National Institute on Aging (Grant NIA U01AG009740) and the Social Security Administration. This analysis uses data or information from the Gateway to Global Aging Data (g2aging.org), produced by the Program on Global Aging, Health & Policy, University of Southern California with funding from the National Institute on Aging (R01AG030153). And the authors disclose the use of DeepSeek‐V3.1 in this work, which was strictly confined to noncreative assistance for language editing and grammar checking.

## Funding

This work was supported by the Humanities and Social Sciences Research Project of Guizhou Provincial Department of Education (Grant 2023GZGXRW150), and the Science and Technology Fund Project of the Guizhou Province Health Commission (Grant gzwkj2024‐548), and the Postdoctoral Startup Project of Zunyi Medical University (Grant F‐ZH‐036).

## Disclosure

All authors critically reviewed and provided feedback on previous versions of the manuscript and approved the final manuscript. It is explicitly stated that all intellectual content and academic insights remain solely attributable to the authors. Any AI‐generated output was critically evaluated and edited prior to use, and the authors take complete responsibility for the entire manuscript.

## Ethics Statement

The data used in this study were retrieved from the Health and Retirement Study (HRS), conducted by the Institute for Social Research at the University of Michigan. The HRS protocol was reviewed and approved by the University of Michigan Health Sciences and Behavioral Sciences Institutional Review Board (IRB HUM00061128). All participants provided written informed consent before each interview wave, and the study was carried out in accordance with the Declaration of Helsinki.

## Conflicts of Interest

The authors declare no conflicts of interest.

## Supporting Information

Additional supporting information can be found online in the Supporting Information section.

## Supporting information


**Supporting Information** Table S1: Sensitivity analysis—subdistribution hazard ratios (SHR) for mortality using Fine–Gray competing‐risk models. Table S2: Subgroup analyses for the association of cumulative depressive symptoms with the risk of cognitive decline. Table S3: Subgroup analyses for the association of cumulative burden with the risk of cognitive decline. Table S4: Subgroup analyses for the association of duration of high exposure with the risk of cognitive decline. Table S5: Subgroup analyses for the association of dynamic change patterns with the risk of cognitive decline. Table S6: Association of cumulative burden and exposure duration (using clinical cut‐point of 3.0) with cognitive decline risk.

## Data Availability

The data that support the findings of this study are available from the Health and Retirement Study data products page (https://hrs.isr.umich.edu/data-products) and the Gateway to Global Aging Data website (https://g2aging.org/).
