# Supplementary material for: Multidimensional Depressive Symptom Exposure and Incident Cognitive Decline: A Prospective Cohort Study
Source: Depress Anxiety. 2026 Aug 6;2026:8846772. doi: 10.1155/da/8846772 (PMC13444727; doi:10.1155/da/8846772)
Supplement: Supplementary file 1 [file DA-2026-8846772-s001.docx]

**Supplementary materials**

**Table S1.** Sensitivity analysis – subdistribution hazard ratios (SHR) for mortality using Fine-Gray competing-risk models

**Table S2.** Subgroup analyses for the association of **cumulative depressive symptoms** with the risk of **cognitive decline**

**Table S3.** Subgroup analyses for the association of **cumulative burden** with the risk of **cognitive decline**

**Table S4.** Subgroup analyses for the association of **duration of high exposure** with the risk of **cognitive decline**

**Table S5.** Subgroup analyses for the association of **dynamic change patterns** with the risk of **cognitive decline**

### **Table S6.** Association of cumulative burden and exposure duration (**using clinical cut-point of 3.0**) with cognitive decline risk

**Table** S**1** Subdistribution hazard ratios (SHR) for mortality – Fine-Gray competing-risk models

| **Exposure** | Model 1 | Model 2 | Model 3 |
| --- | --- | --- | --- |
|  | **SHR (95**%**CI)** | **SHR (95**%**CI)** | **SHR (95**%**CI)** |
| **Cum. expos. (tertiles)** |  |  |  |
| T1 | Reference | Reference | Reference |
| T2 | 1.30 (1.17–1.43) | 1.30 (1.17–1.43) | 1.23 (1.11–1.36) |
| T3 | 1.55 (1.41–1.71) | 1.56 (1.42–1.72) | 1.38 (1.25–1.53) |
| Per 1-unit increase | 1.027 (1.021-1.033) | 1.027 (1.022-1.033) | 1.019 (1.013-1.026) |
| **Cumulative burden** |  |  |  |
| < 0 | Reference | Reference | Reference |
| ≥ 0 | 1.03 (1.02–1.03) | 1.03 (1.02–1.03) | 1.02 (1.01–1.03) |
| **duration of high exposure** |  |  |  |
| **0** | Reference | Reference | Reference |
| **2** | 1.23 (1.11–1.37) | 1.24 (1.12–1.38) | 1.16 (1.04–1.29) |
| **4** | 1.60 (1.37–1.86) | 1.60 (1.38–1.87) | 1.41 (1.21–1.65) |
| **6** | 1.44 (1.22–1.70) | 1.45 (1.23–1.71) | 1.24 (1.05–1.48) |
| **dynamic change patterns** |  |  |  |
| **Reference** | Reference | Reference | Reference |
| Decrease-increase | 1.17 (1.06–1.30) | 1.17 (1.06–1.30) | 1.12 (1.01–1.24) |
| Increase-decrease | 1.24 (1.12–1.37) | 1.24 (1.12–1.37) | 1.16 (1.05–1.29) |
| Increase-increase | 1.58 (1.31–1.91) | 1.58 (1.31–1.91) | 1.43 (1.18–1.73) |

Abbreviations: *SHR*: subdistribution hazard ratios; *CI*: confidence interval.

Model 1: not adjusted;

Model 2: adjusted for age, sex;

Model 3: adjusted for age, sex, education, marital status, smoking status, drinking status, hypertension, diabetes, sleep disorder.

**Table S2** Subgroup analyses for the association of **cumulative depressive symptoms** with the risk of **cognitive decline**

| **Subgroups** | **N** | **T1 (HR 95%CI)** | **T2 (HR 95%CI)** | **T3 (HR 95%CI)** | **P for interaction** |
| --- | --- | --- | --- | --- | --- |
| **Age, years** |  |  |  |  | 0.693 |
| <60 | 1717 | Reference | 1.14 (0.86, 1.52) | 1.38 (1.05, 1.82) |  |
| 60-79 | 5461 | Reference | 1.25 (1.10, 1.41) | 1.44 (1.27, 1.63) |  |
| 80+ | 1669 | Reference | 1.47 (1.15, 1.89) | 1.59 (1.23, 2.06) |  |
| **Sex** |  |  |  |  | 0.017 |
| Male | 3601 | Reference | 1.31 (1.13, 1.53) | 1.23 (1.03, 1.46) |  |
| Female | 5246 | Reference | 1.24 (1.08, 1.43) | 1.59 (1.39, 1.81) |  |
| **Marital status** |  |  |  |  | 0.880 |
| No | 5623 | Reference | 1.28 (1.13, 1.46) | 1.47 (1.29, 1.68) |  |
| Yes | 3224 | Reference | 1.23 (1.04, 1.47) | 1.41 (1.20, 1.66) |  |
| **Smoking** |  |  |  |  | 0.019 |
| No | 4133 | Reference | 1.46 (1.25, 1.70) | 1.67 (1.43, 1.96) |  |
| Yes | 4714 | Reference | 1.13 (0.98, 1.29) | 1.31 (1.14, 1.50) |  |
| **Drinking** |  |  |  |  | 0.344 |
| No | 3442 | Reference | 1.23 (1.05, 1.44) | 1.39 (1.19, 1.63) |  |
| Yes | 5405 | Reference | 1.29 (1.13, 1.47) | 1.52 (1.33, 1.75) |  |
| **Education** |  |  |  |  | 0.699 |
| < High school | 755 | Reference | 1.11 (0.81, 1.52) | 1.26 (0.93, 1.70) |  |
| High school / GED | 2815 | Reference | 1.36 (1.14, 1.61) | 1.49 (1.25, 1.77) |  |
| Bachelor and above | 5277 | Reference | 1.23 (1.07, 1.42) | 1.49 (1.29, 1.71) |  |
| **Sleep disorder** |  |  |  |  | 0.443 |
| No | 7557 | Reference | 1.24 (1.11, 1.38) | 1.45 (1.30, 1.62) |  |
| Yes | 1290 | Reference | 1.53 (1.14, 2.06) | 1.59 (1.21, 2.10) |  |
| **Diabetes** |  |  |  |  | 0.815 |
| No | 6701 | Reference | 1.26 (1.12, 1.42) | 1.49 (1.32, 1.68) |  |
| Yes | 2146 | Reference | 1.27 (1.04, 1.56) | 1.40 (1.15, 1.69) |  |
| **Hypertension** |  |  |  |  | 0.497 |
| No | 3566 | Reference | 1.31 (1.11, 1.55) | 1.57 (1.32, 1.86) |  |
| Yes | 5281 | Reference | 1.23 (1.08, 1.41) | 1.39 (1.23, 1.59) |  |

**Table S3** Subgroup analyses for the association of **cumulative burden** with the risk of **cognitive decline**

| **Subgroups** | **N** | **＜0**  **(HR 95%CI)** | **≥0**  **(HR 95%CI)** | **P for interaction** |
| --- | --- | --- | --- | --- |
| **Age, years** |  |  |  | 0.611 |
| <60 | 1717 | Reference | 1.39 (1.07, 1.80) |  |
| 60-79 | 5461 | Reference | 1.35 (1.19, 1.54) |  |
| 80+ | 1669 | Reference | 1.34 (1.03, 1.73) |  |
| **Sex** |  |  |  | 0.033 |
| Male | 3601 | Reference | 1.12 (0.92, 1.35) |  |
| Female | 5246 | Reference | 1.48 (1.30, 1.68) |  |
| **Marital status** |  |  |  | 0.396 |
| No | 5623 | Reference | 1.40 (1.21, 1.63) |  |
| Yes | 3224 | Reference | 1.30 (1.12, 1.51) |  |
| **Smoking** |  |  |  | 0.208 |
| No | 4133 | Reference | 1.47 (1.25, 1.73) |  |
| Yes | 4714 | Reference | 1.29 (1.13, 1.49) |  |
| **Drinking** |  |  |  | 0.287 |
| No | 3442 | Reference | 1.33 (1.15, 1.55) |  |
| Yes | 5405 | Reference | 1.40 (1.21, 1.62) |  |
| **Education** |  |  |  | 0.260 |
| < High school | 755 | Reference | 1.17 (0.91, 1.52) |  |
| High school / GED | 2815 | Reference | 1.35 (1.13, 1.60) |  |
| Bachelor and above | 5277 | Reference | 1.45 (1.24, 1.69) |  |
| **Sleep disorder** |  |  |  | 0.934 |
| No | 7557 | Reference | 1.36 (1.21, 1.53) |  |
| Yes | 1290 | Reference | 1.36 (1.07, 1.71) |  |
| **Diabetes** |  |  |  | 0.132 |
| No | 6701 | Reference | 1.44 (1.27, 1.64) |  |
| Yes | 2146 | Reference | 1.20 (1.00, 1.45) |  |
| **Hypertension** |  |  |  | 0.179 |
| No | 3566 | Reference | 1.49 (1.25, 1.79) |  |
| Yes | 5281 | Reference | 1.30 (1.14, 1.47) |  |

**Table S4** Subgroup analyses for the association of **duration of high exposure** with the risk of **cognitive decline**

| **Subgroups** | **N** | **0**  **HR (95%CI)** | **2**  **HR (95%CI)** | **4**  **HR (95%CI)** | **6**  **HR (95%CI)** | **P for interaction** |
| --- | --- | --- | --- | --- | --- | --- |
| **Age, years** |  |  |  |  |  | 0.142 |
| <60 | 1717 | Reference | 1.17 (0.90, 1.54) | 1.54 (1.04, 2.27) | 1.24 (0.81, 1.90) |  |
| 60-79 | 5461 | Reference | 1.11 (0.98, 1.27) | 1.50 (1.24, 1.83) | 1.38 (1.12, 1.70) |  |
| 80+ | 1669 | Reference | 1.56 (1.22, 1.98) | 1.37 (0.94, 1.99) | 1.06 (0.66, 1.70) |  |
| **Sex** |  |  |  |  |  | 0.127 |
| Male | 3601 | Reference | 1.07 (0.90, 1.29) | 1.40 (1.05, 1.87) | 0.94 (0.67, 1.33) |  |
| Female | 5246 | Reference | 1.27 (1.11, 1.44) | 1.53 (1.27, 1.85) | 1.50 (1.23, 1.84) |  |
| **Marital status** |  |  |  |  |  | 0.765 |
| No | 5623 | Reference | 1.20 (1.04, 1.39) | 1.59 (1.27, 2.00) | 1.35 (1.04, 1.74) |  |
| Yes | 3224 | Reference | 1.17 (1.00, 1.38) | 1.38 (1.11, 1.72) | 1.26 (1.00, 1.60) |  |
| **Smoking** |  |  |  |  |  | 0.098 |
| No | 4133 | Reference | 1.30 (1.11, 1.53) | 1.85 (1.45, 2.35) | 1.31 (0.97, 1.77) |  |
| Yes | 4714 | Reference | 1.13 (0.98, 1.30) | 1.30 (1.05, 1.59) | 1.30 (1.05, 1.61) |  |
| **Drinking** |  |  |  |  |  | 0.568 |
| No | 3442 | Reference | 1.21 (1.04, 1.42) | 1.53 (1.23, 1.90) | 1.23 (0.96, 1.57) |  |
| Yes | 5405 | Reference | 1.19 (1.03, 1.37) | 1.46 (1.17, 1.83) | 1.43 (1.12, 1.83) |  |
| **Education** |  |  |  |  |  | 0.542 |
| < High school | 755 | Reference | 1.10 (0.83, 1.47) | 1.34 (0.92, 1.96) | 0.97 (0.64, 1.47) |  |
| High school / GED | 2815 | Reference | 1.15 (0.96, 1.37) | 1.47 (1.14, 1.91) | 1.51 (1.15, 1.99) |  |
| Bachelor and above | 5277 | Reference | 1.26 (1.08, 1.46) | 1.53 (1.21, 1.94) | 1.35 (1.04, 1.76) |  |
| **Sleep disorder** |  |  |  |  |  | 0.935 |
| No | 7557 | Reference | 1.20 (1.07, 1.35) | 1.53 (1.28, 1.82) | 1.33 (1.09, 1.63) |  |
| Yes | 1290 | Reference | 1.20 (0.92, 1.57) | 1.32 (0.93, 1.89) | 1.23 (0.87, 1.75) |  |
| **Diabetes** |  |  |  |  |  | 0.398 |
| No | 6701 | Reference | 1.23 (1.08, 1.39) | 1.63 (1.35, 1.97) | 1.39 (1.13, 1.71) |  |
| Yes | 2146 | Reference | 1.14 (0.94, 1.38) | 1.24 (0.94, 1.64) | 1.16 (0.85, 1.59) |  |
| **Hypertension** |  |  |  |  |  | 0.103 |
| No | 3566 | Reference | 1.34 (1.13, 1.60) | 1.85 (1.41, 2.41) | 1.33 (0.96, 1.83) |  |
| Yes | 5281 | Reference | 1.12 (0.98, 1.28) | 1.33 (1.09, 1.61) | 1.30 (1.06, 1.60) |  |

**Table S5** Subgroup analyses for the association of **dynamic change patterns** with the risk of **cognitive decline**

| Subgroups | N | decrease-decrease (HR 95%CI) | decrease-increase (HR 95%CI) | increase-decrease (HR 95%CI) | increase-increase (HR 95%CI) | P for interaction |
| --- | --- | --- | --- | --- | --- | --- |
| **Age, years** |  |  |  |  |  | 0.033 |
| <60 | 1717 | Reference | 1.10 (0.84, 1.44) | 0.90 (0.68, 1.21) | 0.71 (0.39, 1.28) |  |
| 60-79 | 5461 | Reference | 1.10 (0.97, 1.25) | 1.24 (1.10, 1.40) | 1.85 (1.47, 2.31) |  |
| 80+ | 1669 | Reference | 1.36 (1.06, 1.74) | 1.26 (0.98, 1.62) | 1.65 (1.03, 2.63) |  |
| **Sex** |  |  |  |  |  | 0.187 |
| Male | 3601 | Reference | 1.13 (0.96, 1.33) | 1.09 (0.92, 1.29) | 1.15 (0.78, 1.71) |  |
| Female | 5246 | Reference | 1.16 (1.01, 1.33) | 1.26 (1.11, 1.44) | 1.77 (1.41, 2.21) |  |
| **Marital status** |  |  |  |  |  | 0.822 |
| No | 5623 | Reference | 1.14 (0.99, 1.31) | 1.20 (1.05, 1.38) | 1.69 (1.30, 2.18) |  |
| Yes | 3224 | Reference | 1.15 (0.97, 1.35) | 1.17 (1.00, 1.38) | 1.43 (1.07, 1.90) |  |
| **Smoking** |  |  |  |  |  | 0.111 |
| No | 4133 | Reference | 1.22 (1.04, 1.44) | 1.36 (1.16, 1.58) | 1.80 (1.36, 2.39) |  |
| Yes | 4714 | Reference | 1.09 (0.95, 1.26) | 1.08 (0.94, 1.24) | 1.39 (1.07, 1.81) |  |
| **Drinking** |  |  |  |  |  | 0.173 |
| No | 3442 | Reference | 1.12 (0.96, 1.31) | 1.06 (0.90, 1.24) | 1.47 (1.11, 1.95) |  |
| Yes | 5405 | Reference | 1.17 (1.01, 1.35) | 1.30 (1.14, 1.49) | 1.65 (1.27, 2.16) |  |
| **Education** |  |  |  |  |  | 0.729 |
| < High school | 755 | Reference | 1.15 (0.86, 1.54) | 1.10 (0.83, 1.46) | 1.19 (0.70, 2.02) |  |
| High school / GED | 2815 | Reference | 1.15 (0.96, 1.37) | 1.20 (1.01, 1.42) | 1.45 (1.05, 2.00) |  |
| Bachelor and above | 5277 | Reference | 1.14 (0.98, 1.32) | 1.21 (1.04, 1.40) | 1.85 (1.41, 2.42) |  |
| **Sleep disorder** |  |  |  |  |  | 0.635 |
| No | 7557 | Reference | 1.18 (1.05, 1.32) | 1.19 (1.06, 1.33) | 1.53 (1.23, 1.90) |  |
| Yes | 1290 | Reference | 1.01 (0.77, 1.33) | 1.21 (0.93, 1.56) | 1.77 (1.16, 2.71) |  |
| **Diabetes** |  |  |  |  |  | 0.297 |
| No | 6701 | Reference | 1.15 (1.02, 1.31) | 1.25 (1.10, 1.41) | 1.76 (1.40, 2.21) |  |
| Yes | 2146 | Reference | 1.12 (0.92, 1.36) | 1.06 (0.87, 1.29) | 1.24 (0.87, 1.77) |  |
| **Hypertension** |  |  |  |  |  | 0.902 |
| No | 3566 | Reference | 1.10 (0.93, 1.32) | 1.21 (1.02, 1.44) | 1.66 (1.21, 2.27) |  |
| Yes | 5281 | Reference | 1.17 (1.02, 1.33) | 1.18 (1.03, 1.34) | 1.52 (1.20, 1.94) |  |

### **Table S6** Association of cumulative burden and exposure duration (using **clinical cut-point of 3.0**) with cognitive decline risk

| **Exposure** | Case, n(%) | Incidence rate* | Model 1 | Model 2 | Model 3 |
| --- | --- | --- | --- | --- | --- |
|  |  |  | **HR (95**%**CI)** | **HR (95**%**CI)** | **HR (95**%**CI)** |
| **Cumulative burden** |  |  |  |  |  |
| < 0 (n =7,779) | 1958 (25.2) | 6.73 (6.44 - 7.04) | Reference | Reference | Reference |
| ≥ 0 (n = 1,068) | 353 (33.1) | 9.84 (8.84 - 10.9) | 1.43 (1.27-1.60) | 1.44 (1.28-1.61) | 1.27 (1.13-1.43) |
| **Exposure duration** |  |  |  |  |  |
| 0 year (n = 7,091) | 1,749 (24.7) | 6.55 (6.25 - 6.87) | Reference | Reference | Reference |
| 2 years (n = 1,150) | 355 (30.9) | 8.90 (7.99 - 9.87) | 1.33 (1.18-1.49) | 1.35 (1.20-1.51) | 1.23 (1.09-1.38) |
| 4 years (n = 339) | 113 (33.3) | 9.98 (8.23 - 12.0) | 1.48 (1.23-1.80) | 1.50 (1.24-1.81) | 1.32 (1.09-1.60) |
| 6 years (n = 267) | 94 (35.2) | 10.8 (8.73 - 13.2) | 1.57 (1.28-1.94) | 1.61 (1.30-1.98) | 1.34 (1.08-1.65) |
| *P* for trend |  |  | <0.001 | <0.001 | <0.001 |

**Abbreviations:** *HR*: hazard ratio; *CI*: confidence interval.

**Model 1:** not adjusted;

**Model 2:** adjusted for age, sex;

**Model 3:** adjusted for age, sex, education, marital status, smoking status, drinking status, hypertension, diabetes, sleep disorder.

**^*^** Incidence rate per 1000 person-years
